# Characterization Variation of the Differential Coloring Substances in Rapeseed Petals with Different Colors Using UPLC-HESI-MS/MS

**DOI:** 10.3390/molecules28155670

**Published:** 2023-07-26

**Authors:** Haitao Zeng, Tao Zheng, Ying Li, Qiao Chen, Yan Xue, Qi Tang, Hao Xu, Mengjiao Chen

**Affiliations:** 1 Shaanxi Province Key Laboratory of Bio-Resources, Qinba Mountain Area Collaborative Innovation Center of Bioresources Comprehensive Development, Qinba State Key Laboratory of Biological Resources and Ecological Environment (Incubation), School of Biological Science and Engineering, Shaanxi University of Technology, Hanzhong 723001, China; zenghaitao@snut.edu.cn (H.Z.); xh2003@126.com (H.X.);; 2 Hanzhong Institute of Agricultural Sciences, Hanzhong 723001, China; 3 Hanzhong Vocational and Technical College, Hanzhong 723001, China

**Keywords:** rapeseed (*Brassica napus* L.), flavonoids, anthocyanins, petal color, UPLC-HESI-MS/MS

## Abstract

Rapeseed’s (*Brassica napus* L.) colorful petals have important ornamental values. However, the mechanisms of regulating petals coloration in rapeseed are still unknown. In our study, we investigated the key differential coloring substances in nine rapeseed cultivars with different petal colors, and 543 metabolites were detected and characterized through UPLC-HESI-MS/MS. Among them, the kinds and contents of flavonols, flavones, and anthocyanidins were the main contributors to petals’ coloration. Tamarixetin-, quercetin-, butin-, naringenin- and luteolin-derivates were the main pigment bases in white and yellow petals. Peonidin-3,5-*O*-diglucoside, peonidin-3-*O*-(6″-*O*-caffeoyl)glucoside, and quercetin-derivatives were the main coloring substances in pink petals. Acylated cyanidin derivatives might lead to a series of different purple petal colors. Glycosylated anthocyanins were responsible for the coloration of rapeseed red petals, and peonidin-3-*O*-glucoside and kaempferol-derivatives were mainly detected from the red petals. These results provide comprehensive insights into the difference in flavonoid metabolites in rapeseed petals with different colors and supply theoretical supports for the breeding of novel colorful rapeseed cultivars.

## 1. Introduction

Rapeseed (*Brassica napus* L.) petals have bright colors, large petals, long flowering periods, and rich nutrition, which integrate ornamental value and economic value [1,2]. Rapeseed petals have become one of the main flowers in tourism. In nature, the color of rapeseed cultivar petals is yellow. Recently, novel petal color varieties (e.g., pink, orange, red, and purple) have been generated in rapeseed cultivars through interspecific distant hybridization and genetic transformation techniques [3,4]. With the vigorous development of rapeseed flower tourism, the breeding of rapeseed cultivars with different petal colors is one of the key objects of rapeseed germplasm resource innovation and genetic breeding. However, the mechanisms of regulating petal color in rapeseed are still unclear. Thus, it is of great potential to breed rapeseed with specific flower colors, and understanding the variation of flavonoids in rapeseed germplasm will be beneficial to the efficient breeding of new rapeseed cultivars with richer flower colors and higher ornamental values.

Plant petals are the vital parts of the corolla, and petal color is not only one of the important ornamental traits of plants, but also has the function of attracting insect pollination and reproducing offspring. The natural coloring substances that influence the color formation of plant petals are mainly flavonoids, carotenoids, and alkaloids [5]. The types and contents of those three pigments directly or indirectly affect the petals’ color [6,7]. Flavonoid pigments are divided into flavonoids, flavonols, flavanones, flavanols, anthocyanins, and isoflavones [8,9]. The flavonoid pigments contribute to making plants presented with a variety of flower colors, including pink, red, purple, blue, and yellow [10]. Anthocyanins are extremely key pigments in plants that regulate the color formation of fruits, leaves, and petals [11], and anthocyanins could lead the petals and fruits of plants to present with a variety of colors, such as blue, purple, orange, yellow, and red [12]. Chalcone and aurone often appear in the same petal and show dark yellow, and flavonoids and flavonols are colorless or pale yellow [13], which can form complexes with anthocyanins to form more complex structures, thus affecting flower color [14]. Anthocyanins are the main coloring substance in plants, and various factors such as the type of anthocyanin or the anabolic pathway also have an important influence on flower coloration [15]. Anthocyanins can be divided into three types: pelargonidin, delphinidin, and cyanin, which exist alone in different flowers, showing a brick red, a bluish-purple, and a purplish-red color, respectively [16]. Peonidin-, petunidin-, and malvidin-pigments contribute to red or purple colors, among which peonidin is responsible for a purplish-red color, and petunidin- and malvidin-derivatives are responsible for a bluish-purple color [17,18]. A mass of flavonoids and anthocyanins have been isolated and detected from *Brassica* vegetables in recent years, such as flavonoids, flavonols, flavones, aurones, and anthocyanins with specified chemical structures [19]. Correspondingly, 130 flavonol glycosides have been successfully isolated and identified in more than 30 *Brassica* vegetables, and 50 anthocyanins with different glycoside units have been found in Brassica vegetables with different colors [20]. Lin et al. (2011) have determined 102 flavonol glycosides and 67 anthocyanins in the red leaves of *Brassica juncea* [21]. However, the specific pigments regulating the rapeseed petals’ coloration and the differences in chemical composition in rapeseed with different colors are still unknown.

Metabolomics technology is widely used in plant nutrition, botanical sciences, stress biology, and plant metabolism, using the qualitative and quantitative analysis of small molecule metabolites in a particular tissue of a plant through high-throughput chemical analysis technology [19]. In particular, ultrahigh-performance liquid chromatography-HESI-mass spectrometry (UPLC-HESI-MS/MS), a rapid and highly powerful method, is widely studied and applied to detect flavonoids compounds and anthocyanins in many plant petals, such as *Chenopodium quinoa* Wild. [22], *Brassica campestris ssp pekinensis* [23], cabbage petals [24], *Camellia reticulata* [25], and *Rhododendron latoucheae* [26]. Therefore, investigating the functional compounds that regulate flower coloration using UPLC-HESI-MS/MS could gain insights into the diversity of differential metabolites in rapeseed petals with different colors and is helpful in understanding the material basis of rapeseed flower color formation.

In our study, to understand the main metabolic components in rapeseed petals and compare the differential components among petals with different colors, the UPLC-HESI-MS/MS technology was performed to assess the differential coloring compounds in rapeseed petals with different colors in the same habitat. And, we characterized the differential accumulated compounds between typical petals’ color (white and yellow) and the colored petals (pink, purple, and red) using hierarchical cluster analysis (HCA), principal component analysis (PCA), and orthogonal partial least squares discriminant analysis (OPLS-DA). Thus, our results could improve the understanding of the metabolic differences between flavonoid compounds and anthocyanins in rapeseed petals with different colors, thereby revealing the material basis for the formation of different-colored rapeseed petals, providing convenience for the selective breeding of colorful rapeseed cultivars, and helping the healthy development of the Chinese rapeseed ornamental tourism industry.

## 2. Results

### 2.1. UPLC-HESI-MS/MS Analysis of Metabolites Profiling in Rapeseed Petals with Different Colors

**Figure 1:**
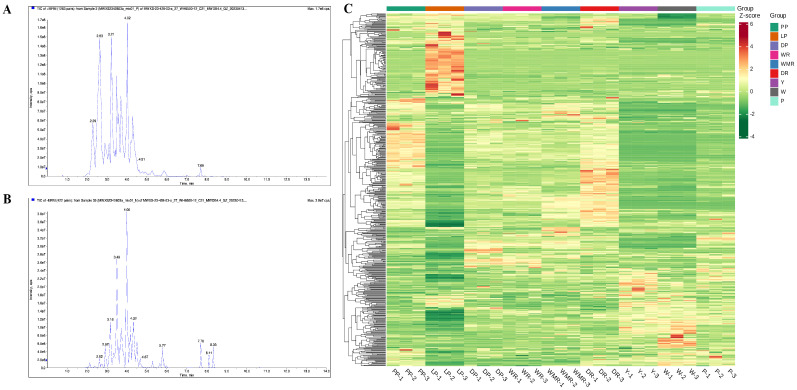
Mass spectrometry analysis of QC (quality control) samples total ion current diagram. (**A**) The total ion current curves of the positive ion mode (P+). (**B**) The total ion current diagram of the negative ion mode (N−). The horizontal coordinate represented the retention time (Rt) of metabolite detection, and the vertical coordinate represented the ion current intensity of ion detection (intensity unit is cps, count per second). (**C**) Hierarchical clustering heat map of 543 metabolites in 9 rapeseed petals. The tree map on the left side of the heat map represented the clustering results of the 543 metabolites; different colors in the heat map represented the relative content of each metabolite, of which red color indicated the metabolite with high content, and green color indicated the metabolite with low content.

The UPLC-HESI-MS/MS method was carried out to detect and analyze the metabolite profiling in nine rapeseed petals with different flower colors. The total ion current curves of the positive ion mode (P+) (Figure 1A) and the total ion current diagram of the negative ion mode (N−) (Figure 1B) of the mass spectrometry analysis were highly coincident, indicating that the signal stability of the UPLC-HESI-MS/MS detection system was excellent. Based on Met Ware’s self-built databases, five hundred and forty three metabolites were determined and identified in nine petals with different colors (Table S1), which could be divided into ten main groups: eight tannins, thirteen flavanols, one hundred and fifty eight flavonols, one hundred and forty seven flavones, one hundred and four anthocyanidins, thirteen flavanonols, fifty one flavanones, three aurones, twenty four chalcones, and twenty two other flavonoids, which indicated that the important substances responsible for different color formations in rapeseed petals were predominantly engaged in flavonols, flavones, and anthocyanidins.

The results of the heatmap clusters of nine rapeseed petals samples were established on the basis of the five hundred and forty three metabolites contents, which obviously demonstrated that samples from the same color clustered together (top side of Figure 1C), suggesting that the metabolite data had high accuracy and reliability. Moreover, all the metabolites were grouped into four main groups exhibiting differential accumulation levels between yellow, pink, purple, and red color petals samples. As shown in Figure S1, the contribution rates of PC1 and PC2 were 30.92% and 22.35%, respectively, and the cumulative contribution rate reached 53.27%. A clear separation was readily observed between the different-colored rapeseed petals samples in the 2D-PCA score plot, indicating that the metabolites in the different-colored petals were significantly distinct, and three biological repeats of each petal with the same color were tightly gathered in a compact cluster. The PCA results suggested that the metabolites in the nine rapeseed petals with different colors were significantly distinct, and the difference between the sample repetitions was small, indicating that the metabolomics data could be used for subsequent difference analysis.

### 2.2. Differential Accumulated Metabolites between Yellow and White Petals in Rapeseed

In white and yellow rapeseed petals, a total of 520 metabolites were identified. To further reveal the differential metabolites between white and yellow petals, the metabolites between W and Y petal samples were analyzed, and 26 significantly high numbers of differential accumulated metabolites (DAMs) were screened out (Table S2), among which there were 14 up-accumulated and 12 down-accumulated DAMs among the W and Y petal samples, of which there were mainly flavonols and flavones.

Further, we investigated the variations of the most significant flavonols and flavones in the W and Y petals. It was found that tamarixetin-3-*O*-rutinoside, quercetin-3,7-Di-*O*-rhamnoside, butin-7-*O*-glucoside, isorhamnetin-3-*O*-rutinoside, and naringenin-7-*O*-glucoside displayed a high accumulation level in the W petals (Figure S2), among which tamarixetin-3-*O*-rutinoside and isorhamnetin-3-*O*-rutinoside were down-regulated by 4.35 × 10^−1^- and 2.45 × 10^−5^-fold, respectively. Kaempferol-3-*O*-(6″-malonyl)sophorotrioside, 3,5,4′-Trihydroxy-7-methoxyflavone, quercetagetin, tamarixetin, isorhamnetin, luteolin-7-*O*-glucoside, kaempferol-3-*O*-galactoside, tricin-5,7-*O*-diglucoside, phellodendroside, and 4′-hydroxy-2,4,6-trimethoxydihydrochalcone exhibited a high yield in the Y petals compared with the W petals, of which tamarixetin and 4′-hydroxy-2,4,6-trimethoxydihydrochalcone were up-regulated by 3.29 × 10^0^- and 3.19 × 10^0^-fold, respectively.

Accordingly, the tamarixetin-3-*O*-rutinoside and isorhamnetin-3-*O*-rutinoside might be the key metabolites contributing to white color formation in rapeseed petals. Studies have shown that chalcone could regulate the formation of deep yellow petals [13]. In yellow rapeseed petals, tamarixetin and 4′-hydroxy-2,4,6-trimethoxydihydrochalcone increased significantly in comparison with white petals, suggesting that tamarixetin and 4′-hydroxy-2,4,6-trimethoxydihydrochalcone might be closely related to the color formation of the yellow rapeseed petals. Therefore, we could breed specific rapeseed cultivars with different-colored petals by monitoring the metabolomic variations.

### 2.3. Differential Accumulated Metabolites in Rapeseed Pink Petals

There were 537 metabolites identified between white- and pink-colored rapeseed petals, and 137 metabolites (123 upregulated and 14 downregulated) were selected as the significantly differential flavonoids between the white and pink petals (Table S3). Most flavonoids were present with higher accumulation levels in the P petals than those in the W petals. In pink rapeseed petals, the differentially accumulated metabolites were mainly concentrated in flavonols and anthocyanins compared with white petals.

**Figure 2:**
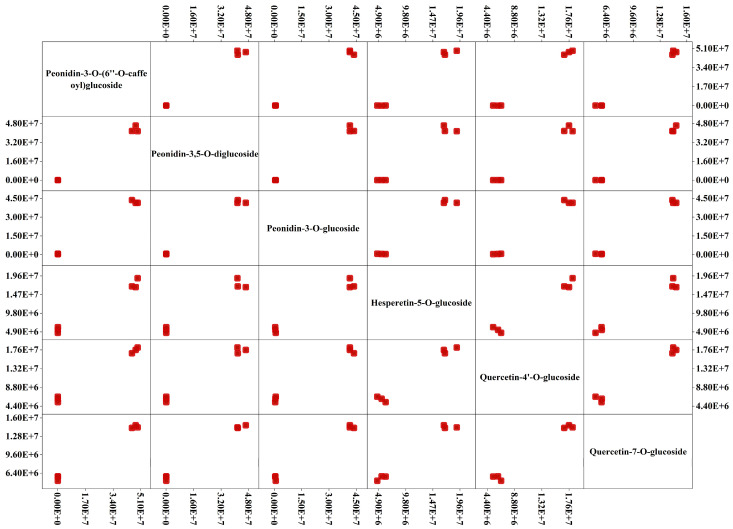
Scatter matrix analysis of the most significantly different substances in W and P petals. The red squares represent the peak areas of different metabolites. The horizontal coordinate and the vertical coordinate represent the peak areas’ values.

The higher concentrations in the P petals were peonidin glycosides (peonidin-3-*O*-(6″-*O*-caffeoyl)glucoside, peonidin-3,5-*O*-diglucoside, peonidin-3-*O*-glucoside), quercetin-4′-*O*-glucoside, hesperetin-5-*O*-glucoside, and quercetin-7-*O*-glucoside, which were up-regulated by 4.02 × 10^2^-, 4.71 × 10^2^-, 7.38 × 10^1^-, 2.91 × 10^0^-, 3.25 × 10^0^-, and 3.25 × 10^0^-fold, respectively, compared with the W petals (Figure 2). These results indicate that the contents of peonidin-3,5-*O*-diglucoside, quercetin-4′-*O*-glucoside, hesperetin-5-*O*-glucoside, and quercetin-7-*O*-glucoside were the main contributors to pink rapeseed petals, and peonidin-3,5-*O*-diglucoside was the most important anthocyanin component in pink rapeseed petals, which might be the cause of pink petals. Thus, it could be seen that to keep the petals of rapeseed bright pink, it was necessary to increase the content of peonidin-3,5-*O*-diglucoside, quercetin-4′-*O*-glucoside, hesperetin-5-*O*-glucoside, and quercetin-7-*O*-glucoside.

### 2.4. Differential Accumulated Metabolites between Light Purple, Purple, and Dark Purple Petals in Rapeseed

**Figure 3:**
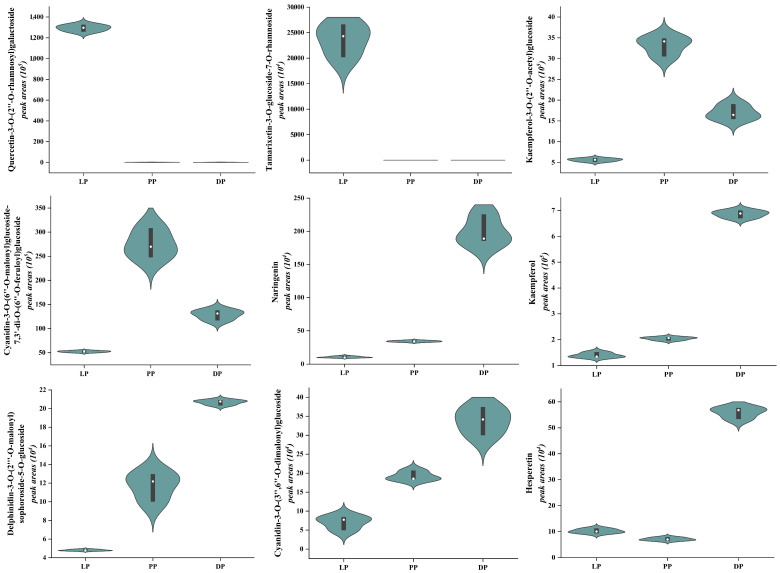
Violin plots of peak area values of nine differentially accumulated metabolites identified in light purple, purple, and dark purple petals. The distribution and probability density of the nine major DAMs were represented by a combination of box plots and density plots. The outer shapes represented the density of the peak area value distribution, the black rectangular box in the middle represented the quartile range, and the white circle in the middle was the median.

In our study, we detected 348 DAMs between LP_vs_PP, LP_vs_DP, and PP_vs_DP; the common differential metabolites in the three comparisons were among 30 DAMs (Figure S3A). The anthocyanidins, flavones, flavanones, and flavonols were the main contributors to different purple rapeseed petals (Table S4), among which the cyanidin derivatives exhibited the highest accumulation and were the main coloring substances, such as cyanidin-3-*O*-[2-(2-(sinapoyl)-xylosyl)-6-(caffeoyl)-glucoside]-5-*O*-[6-(malonyl)glucoside, cyanidin-3-diferuloyl-diglucoside-5-malonylglucoside, cyanidin-3-*O*-(6″-*O*-malonyl)glucoside-7,3′-di-*O*-(6″-*O*-feruloyl)glucoside, and cyanidin-3-*O*-(6″-Osinapoyl-2″-*O*-malonyl)glucoside-5-*O*-(6‴-*O*-malonyl)glucoside. In the comparison of LP_vs_ PP, cyanidin-3-*O*-[2-(2-(sinapoyl)-xylosyl)-6-(caffeoyl)-glucoside]-5-*O*-[6-(malonyl)-glucoside], cyanidin-3-*O*-(6″-*O*-malonyl)glucoside-7,3′-di-*O*-(6″-*O*-feruloyl)glucoside, cyanidin-3,5-*O*-diglucoside, and kaempferol-3-*O*-(2″-*O*-acetyl)glucoside were up-regulated by 4.67 × 10^0^-, 5.26 × 10^0^-, 2.87 × 10^0^-, and 6.69 × 10^0^-fold, respectively, and tamarixetin-3-*O*-glucoside-7-*O*-rhamnoside, quercetin-3-*O*-(2″-*O*-rhamnosyl)galactoside, quercetin-3-*O*-(2″-*O*-Rhamnosyl)rutinoside, and delphin-idin-3-*O*-(2‴-*O*-p-coumaroyl)rutinoside were down-regulated by 1.31 × 10^−4^-, 1.54 × 10^−3^-, 3.45 × 10^−4^- and 3.45 × 10^−4^-fold, respectively. In the comparison of LP_vs_ DP, hesperetin, cyanidin-3-*O*-(3″,6″-*O*-dimalonyl)glucoside, delphinidin-3-*O*-(2‴-*O*-malonyl)sophoroside-5-*O*-glucoside, naringenin, hesperetin-5-*O*-glucoside, and catechin were ur-accumulated by 5.47 × 10^0^-, 4.81 × 10^0^-, 2.56 × 10^0^-, 1.89 × 10^1^-, 2.67 × 10^0^-, and 2.01 × 10^0^-fold, respectively, and tamarixetin-3-*O*-glucoside-7-*O*-rhamnoside and quercetin-3-*O*-(2″-*O*-rhamnosyl)galactoside were down-regulated by 1.57 × 10^−4^- and 1.58 × 10^−3^-fold. In the comparison of PP_vs_ DP, naringenin, hesperetin, catechin, kaempferol, delphinidin-3-*O*-(2‴-*O*-malonyl)sophoroside-5-*O*-glucoside, and cyanidin-3-*O*-(3″,6″-*O*-dimalonyl)glucoside were up-accumulated by 5.87 × 10^0^-, 7.86 × 10^0^-, 3.19 × 10^0^-, 3.36 × 10^0^-, 1.10 × 10^0^-, and 1.76 × 10^−0^-fold, respectively, and cyanidin-3-*O*-(6″-*O*-m-alonyl)glucoside-7,3′-di-*O*-(6″-*O*-feruloyl)glucoside and kaempferol-3-*O*-(2″-*O*-acetyl)gluco-side were down-regulated by 4.68 × 10^−1^- and 4.12 × 10^−1^-fold, respectively. Therefore, tamarixetin-3-*O*-glucoside-7-*O*-rhamnoside and quercetin-3-*O*-(2″-*O*-rhamnosyl)galactoside were the main color constituents in light purple petals, cyanidin-3-*O*-(6″-*O*-malonyl)glucoside-7,3′-d-i-*O*-(6″-*O*-feruloyl)glucoside and kaempferol-3-*O*-(2″-*O*-acetyl)glucoside were the important color substances responsible for the purple petals, and delphinidin-3-*O*-(2‴-*O*-malonyl)sophorosi-de-5-*O*-glucoside, naringenin, hesperetin, catechin, kaempferol, and cyanidin-3-*O*-(3″,6″-*O*-dimalonyl)glucoside were the main color constituents in dark purple petals (Figure 3). Quercetin, cyanidin, and delphinidin derivatives were the main coloring substances in purple petals. Flavonols play important roles in the formation of blue or purple–red series, and the auxiliary effect between kaempferol derivatives and cyanidins might be the key formation mechanism of purple petals in rapeseed. These results suggest the DAMs above acted as part of the pigment composition in light purple, purple, and dark purple petals.

### 2.5. Differential Accumulated Metabolites between Wine Red, Watermelon Red, and Dark Red Petals in Rapeseed

**Figure 4:**
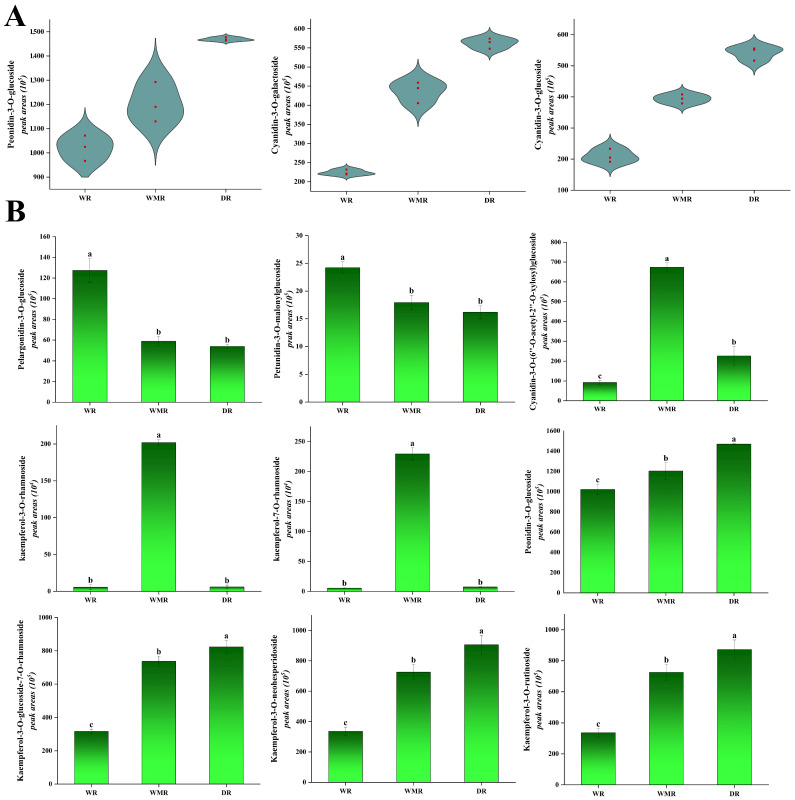
(**A**) Violin plots of peak areas values of three main anthocyanins in wine red, watermelon red, and dark red petals. The outer shapes represented the density of the peak area value distribution; the red rectangular box represented the peak area values. (**B**) Histogram of peak areas of nine differentially accumulated metabolites identified in wine red, watermelon red, and dark red petals. Duncan’s test was applied to evaluate the significant difference among petals with different colors. The different letters in the histogram represented the significant difference at *p* < 0.05 level.

In total, 541 metabolites were determined and characterized in wine red, watermelon red, and dark red rapeseed petals, of which 104 anthocyanins were determined and identified. Peonidin-3-*O*-glucoside, cyanidin-3-*O*-galactoside, and cyanidin-3-*O*-glucoside were the main anthocyanins contributing to red petals (Figure 4A). A amount of 181 significantly accumulated flavonoid compounds were identified in the comparisons LP_vs_PP, LP_vs_DP, and PP_vs_DP (Table S5). The differential metabolites in the petals of the three red colors were significantly enriched in the flavonoid biosynthesis and anthocyanins biosynthesis, indicating that the difference in the content of flavonoid and anthocyanins metabolites may affect the petals color in rapeseed red lines.

Among WR, WMR, and DR petals, there were five common compounds, including cyanidin-3-*O*-(6″-*O*-acetyl-2″-*O*-xylosyl)glucoside, tamarixetin-3-*O*-gluco-side-7-*O*-sulon-ate, kaempferol-3-*O*-(2‴-p-Coumaroyl)sophoros-ide-7-*O*-Glucoside, okanin-3′-*O*-β-d-glucoside, and kaempferol-3-*O*-(6″-*O*-acetyl)glucoside (Figure S3B). In the comparison of WR_vs_WMR, kaempferol-7-*O*-rhamnoside, kaempferol-3-*O*-rhamn-oside, and cyanidin-3-*O*-(6″-*O*-acetyl-2″-*O*-xylosyl)glucoside were up-regulated by 4.07 × 10^1^-, 3.61 × 10^0^-, and 7.29 × 10^0^-fold, respectively, whereas pelargonidin-3-*O*-glucoside and petunidin-3-*O*-malonylglucoside were down-regulated 4.60 × 10^−1^- and 3.45 × 10^−1^-fold, respectively (Figure 4B). In the comparison of WR_vs_DR, cyanidin-3-*O*-galactoside, cyanidin-3-*O*-glucoside, kaempferol-3-*O*-glucoside-7-*O*-rhamnoside, kaempferol-3-*O*-neohesperidoside, and kaempferol-3-*O*-rutinoside were up-regulated by 2.21 × 10^0^-, 2.58 × 10^0^-, 2.60 × 10^0^-, 2.60 × 10^0^-, and 2.60 × 10^0^-fold, respectively, while pelargonidin-3-*O*-glucoside and petunidin-3-*O*-glucoside were down-regulated 4.22 × 10^−1^ and 4.64 × 10^−1^-fold, respectively. In the comparison of WMR_vs_DR, cyanidin-3-*O*-(6″-*O*-acetyl-2″-*O*-xylosyl)glucoside, kaempferol-3-*O*-rhamnoside, and kaempferol-7-*O*-rhamnoside were down-regulated by 3.37 × 10^−1^, 2.98 × 10^−2^, and 3.24 × 10^−2^-fold, respectively. Therefore, pelargonidin-3-*O*-glucoside and petunidin-3-*O*-malonylglucoside were the main compounds contributing to the wine red petals; cyanidin-3-*O*-(6″-*O*-acetyl-2″-*O*-xylosyl)glucoside, kaempferol-3-*O*-rhamnoside, and kaempferol-7-*O*-rhamnoside were responsible for the watermelon red color formation; and peonidin-3-*O*-glucoside, kaempferol-3-*O*-rutinoside, kaempferol-3-*O*-glucoside-7-*O*-rhamnoside, and kaempferol-3-*O*-neohesperidoside were the main substances that led to dark red petals (Figure 4B). The more peonidin-3-*O*-glucoside, cyanidin-3-*O*-galactoside, and cyanidin-3-*O*-glucoside existed in the vacuole, the redder the petals displayed, and the red effect of peonidin-3-*O*-glucoside was more significant. The factors that aroused color variation in the red petals were considered to be kaempferol derivatives. Consequently, we conjectured that peonidin-3-*O*-glucoside, cyanidin-3-*O*-galactoside, cyanidin-3-*O*-glucoside, and kaempferol derivatives could act as the co-pigments in wine red, watermelon red, and dark red petals.

### 2.6. Differential Accumulated Metabolites between White, Yellow, Purple, and Red Petals in Rapeseed

**Figure 5:**
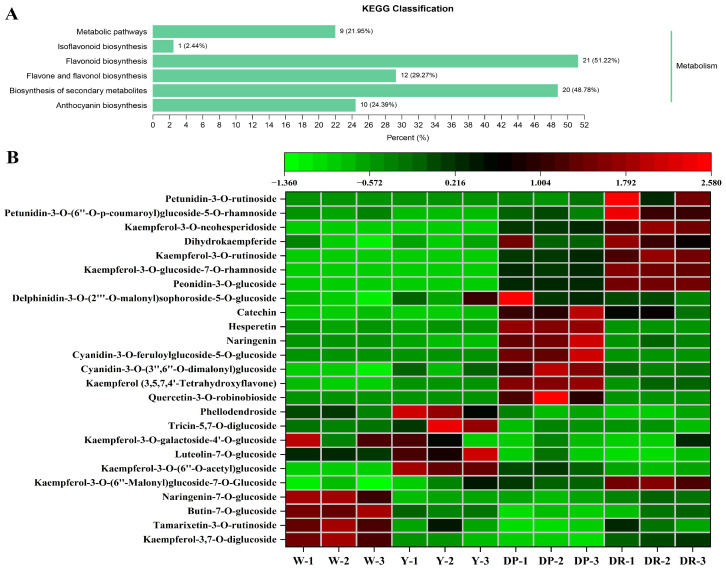
(**A**) The differential KEGG pathways in W, Y, DP, and DR petals. (**B**) Heatmap analysis of the compounds differentially accumulated in W, Y, DP, and DR petals. The green color indicates a low accumulation level of each metabolite and the red color indicates a high accumulation level of each metabolite.

In our study, 249 differentially accumulated compounds were screened out between W, Y, DP, and DR petals, including 63 flavonols, 54 flavones, 73 anthocyanins, 21 flavanones, and others (Table S6). The KEGG pathway database is a useful tool for the annotation analysis and enrichment analysis of the differential accumulated metabolites, which implemented the visual representation of different metabolic pathways. All the differentially accumulated metabolites in the rapeseed petal samples with different red colors were mapped to KEGG, and the main differential metabolic pathways between different petals samples were obtained. Our results exhibited that the top three metabolic pathways were associated with the flavonoid biosynthesis pathway, the flavone and flavonol biosynthesis pathway, and the anthocyanin biosynthesis pathway (Figure 5A).

As expected, kaempferol-3,7-*O*-diglucoside, tamarixetin-3-*O*-rutinoside, butin-7-*O*-glucoside, and naringenin-7-*O*-glucoside were the main compounds in the W petals (Figure 5B). Kaempferol-3-*O*-(6″-malonyl)sophorotrioside, luteolin-7-*O*-glucoside, kaempferol-3-*O*-galactoside, tricin-5,7-*O*-diglucoside, and phellodendroside were the main accumulated substances in the yellow petals. In the comparison Y_vs_DP, there were 25 DAMs (11 up-regulated/14 down-regulated). Quercetin-3-*O*-robinobioside, kaempferol, cyanidin-3-*O*-(3″,6″-*O*-dimalonyl)glucoside, cyanidin-3-*O*-feruloylglucoside-5-*O*-glucoside, naringenin, hesperetin, catechin, and delphinidin-3-*O*-(2‴-*O*-malonyl)sophoroside-5-*O*-glucoside were the main color constituents in dark purple petals, among which cyanidin-3-*O*-(6″-*O*-caffeoyl)sophoroside-7-*O*-glucoside and cyanidin-3-*O*-feruloylglucoside-5-*O*-glucoside were significantly up-regulated by 2.39 × 10^5^- and 2.32 × 10^5^-fold, respectively. In the comparison DP_vs_DR, there were 52 up-regulated DAMs and 25 down-regulated DAMs. Peonidin-3-*O*-glucoside, kaempferol-3-*O*-glucoside-7-*O*-rhamnoside, kaempferol-3-*O*-rutinoside, dihydrokaempferide, kaempferol-3-*O*-neohesperidoside, petunidin-3-*O*-(6″-*O*-p-coumaroyl)glucoside-5-*O*-rhamnoside, and petunidin-3-*O*-rutinoside were the main substances that led to dark red petals, among which peonidin-3-*O*-glucoside and kaempferol-3-*O*-glucoside-7-*O*-rhamnoside were up-regulated by 2.03 × 10^0^-, and 2.09 × 10^0^-fold, respectively. In addition, the content of cyanidin-3-*O*-feruloylglucoside-5-*O*-glucoside in the DP petals was down-regulated by 4.32 × 10^−6^-fold, compared with that in the DR petals.

These findings indicate that the main metabolites above were markedly different between the rapeseed petals with different colors, of which the tamarixetin-3-*O*-rutinoside was the key factor for white color formation, tamarixetin and 4’-hydroxy-2,4,6-trimethoxydihydrochalcone were the main coloring substances in yellow rapeseed petals, cyanidin-3-*O*-(6″-*O*-caffeoyl)sophoroside-7-*O*-glucoside and cyanidin-3-*O*-feruloylglucoside-5-*O*-glucoside were the key metabolites for dark purple coloration in rapeseed petals, and peonidin-3-*O*-glucoside and kaempferol-3-*O*-glucoside-7-*O*-rhamnoside were the main anthocyanins contributing to dark red coloration.

## 3. Discussion

Petal color is one of the important traits of ornamental plants, and it is also one of the main criteria for people to select and appreciate plants [27]. Flower color could protect plants from diseases and ultraviolet radiation and help maintain normal physiological functions, such as flower organ differentiation [28]. For ornamental plants, plant petal color plays an important role in their ornamental and commercial value [29]. Recently, tourists have been attracted by the colorful rapeseed petals. As a common economic crop in China, rapeseed has a long history of cultivation and has produced a rich rape culture [1]. In general, the colors of rapeseed petals are mostly yellow, lesser white, milky white, and orange. In recent years, colorful rapeseed flowers have been bred in rapeseed cultivars, which greatly enhances the ornamental value of rapeseed flowers [2]. In rapeseed, previous studies have focused on the oil accumulation and high yield [30], and the breeding of novel rapeseed varieties [31], whereas the research on the metabolic distinctions and the differential compounds in rapeseed petals with different colors are seldom reported.

Flavonoids are a large class of secondary metabolites in plants, especially in the petals of plants, and are important substances for the coloration of plant petals, of which the differences in their types and contents leads to the formation of petals with different colors [32]. Flavones and flavonols were only found in the white *Chrysanthemum* petals, whereas anthocyanins, flavones, and flavonols were rich in the pink *Chrysanthemum* petals [28]. Flavones and flavonols, as important co-pigments of anthocyanins, also have important effects on the variation and improvement of flower color [33]. Fossen et al. (2000) found that flavonols and anthocyanins were connected to form new reconstructed anthocyanins through glucosyl residues in their respective structures in the light purple petals of *Allium schoenoprasum* [34]. Heursel (1981) found that the blue-toned flower color appeared in the hybrid offspring of *Rhododendron simsii*, which was caused by the presence of flavonols [35]. Peonidin-3-*O*-glucoside and delphinidin-3-*O*-glucoside were the main coloring substances in the red and red-purple petals of *Catharanthus roseus* varieties, respectively, and phenolic compounds, especially flavonoids such as hesperetin, rutin, and liquiritigenin, could be used as auxiliary pigments to assist in coloring [36]. Anthocyanins are key metabolites responsible for plant organs’ coloration, contributing to the generation of different petal colors. Anthocyanin metabolites are essential for petals’ colors, and there is a great deal of metabolic variability among different-colored petals. Correspondingly, kaempferol-3-*O*-(2″ acetyl)glucoside and 5,4’-Dihydroxy-7-methoxyflavanone, tricetin, homoeriodictyol, apigenin, and sakura were identified as the differential accumulated metabolites in *Brassica campestris* ssp. pekinensis, which were responsible for the coloration of yellow and white petals [37]. Yin et al. (2019) investigated the differential coloring substances in four rapeseed petals with different colors, and kaempferol derivates were mainly in pale white and yellow petals; epicatechin, quercetin, and isorhamnetin derivates mainly existed in pink and red petals. In addition, cyanidin-3-*O*-glucoside, cyanidin-3,5-*O*-diglucoside, and petunidin-3-*O*-glucoside were rich in red rapeseed petals [1]. In white quinoa grains cultivars, the significant accumulation of the tamarixetin derivatives (tamarixetin-3-*O*-rutinoside, tamarixetin-3-*O*-glucoside-7-*O*-rhamnoside) and isorhamnetin-3-*O*-rutinoside were observed; kaempferol derivatives displayed a higher accumulation in red quinoa grains cultivars [22]. The difference in anthocyanin content has a direct effect on the color of plant petals. Therefore, the qualitative and quantitative analysis of flavonoids and anthocyanins in the white, yellow, pink, purple, and red color petals of rapeseed cultivars was carried out using UPLC-HESI-MS/MS in the present study.

We analyzed the flavonoid and anthocyanin compounds in nine rapeseed cultivars with different petal colors, and found that the chemical compounds in petals with different colors were obviously different. In the white rapeseed petals, it was found that the butin-7-*O*-glucoside, naringenin-7-*O*-glucoside, isorhamnetin-3-*O*-rutinoside, and tamarixetin-3-*O*-rutinoside had higher levels in white petals, which was in concordance with the results that color change in quinoa grains was linked to flavone and flavonol biosynthesis [22]. In yellow rapeseed petals, tamarixetin, 4’-hydroxy-2,4,6-trimethoxydihydrochalcone, luteolin-7-*O*-glucoside, kaempferol-3-*O*-(6″-malonyl)sophorotrioside, kaempferol-3-*O*-galactoside, tricin-5,7-*O*-diglucoside, and phellodendroside were the main accumulated substances, which was consistent with this conclusion that chalcone could regulate the formation of deep yellow petals [13], indicating that tamarixetin and 4′-hydroxy-2,4,6-trimethoxydihydrochalcone were the main contributors to yellow petals. In addition, trace amounts of malvidin and delphinidin were detected in white and yellow rapeseed petals, respectively, but the petals did not show blue or purple, mainly due to the coloration of the eye part. In rapeseed pink petals, the hesperetin- and quercetin-derivative compounds, peonidin-3,5-*O*-diglucoside and peonidin-3-*O*-(6″-*O*-caffeoyl)glucoside were found at a higher level in comparison with the white petals, which was in accordance with the results that peonidin-3,5-*O*-diglucoside and peonidin-3-*O*-(6″-*O*-caffeoyl)glucoside were the main pigments in *Camellia japonica* pink petals [12].

In purple and red rapeseed petals, the contents of cyanidin, delphinidin, peonidin, and kaempferol derivatives were present at higher levels than those in yellow and white petals, which is consistent with the results that color variations are related to anthocyanin biosynthesis in strawberry and *Catharanthus roseus* [9]. Previous studies revealed that cyanidin, delphinidin, petunidin, and pelargonidin glycosides were the main anthocyanins in petal coloration in many plants, such as *Camellia sinensis* [25], grape [38], *Lycium ruthenicum* Murray [39], and strawberry [40]. Additionally, anthocyanins have a series of ways to modify themselves, including glycosylation, acylation, and methylation. The glycosylation and methylation of anthocyanins could contribute to the petals’ redder color, acylation modification could change the absorption wavelength of anthocyanins and affect the petals’ color; for example, acylation modification might make the plant color purple, and acylated anthocyanins are mainly found in dark vegetables and tubers, such as *Ipomoea batatas*, *Solanum tuberdsm*, and purple Cabbage [41]. Here, we observed similar results that cyanidin-3-diferuloyl-diglucoside-5-malonylglucoside, cyanidin-3-*O*-[2-(2-(sinapoyl)-xylosyl)-6-(caffeoyl)-glucoside]-5-*O*-[6-(malonyl)glucoside], cyanidin-3-*O*-(6″-*O*-malonyl)glucoside-7,3′-di-*O*-(6″-*O*-feruloyl)glucoside, cyanid-in-3-*O*-(6″-*O*-sinapoyl-2″-*O*-malonyl)glucoside-5-*O*-(6‴-*O*-malonyl)glucoside, cyanidin-3-*O*-ferulo-ylglucoside-5-*O*-glucoside, cyanidin-3-*O*-(3″,6″-*O*-dimalonyl)glucoside, and delphinidin-3-*O*-(2‴-*O*-malonyl)sophoroside-5-*O*-glucoside were the main anthocyanins in purple petals, and all of them belonged to acylated anthocyanins. The composition and content of flavonols also have a certain effect on petal colors, which at least contribute to pigmentation, and lead to a bluish-purple effect and also to darker colors. In the purple petals of rapeseed, the flavonol composition patterns were mainly made up of quercetin derivatives.

In red rapeseed petals, peonidin-3-*O*-glucoside, cyanidin-3-*O*-galactoside, and cyanidin-3-*O*-glucoside were found at higher levels, which belong to glycosylated anthocyanins, suggesting that those anthocyanins might be responsible for the coloration of red rapeseed petals, which is consistent with the results that glycosylated anthocyanins were the key pigments in red carnation flowers [42]. Kaempferol derivatives are seldom reported to be related to the petal coloration in other horticultural ornamental plants, whereas the kaempferol derivatives displayed markedly high accumulation levels in red rapeseed petals, demonstrating that the kaempferol derivatives in rapeseed are obviously different than those in other floricultural plants. However, Yin et al. (2019) found that kaempferol derivates were mainly detected in pale white and yellow rapeseed petals, which was different from our results [1]. The reason for the difference in results might be that the rapeseed petals in our research had different genetic backgrounds from the petals performed in prior research. Hence, more biomolecular research is needed to explore and confirm the coloration mechanism of rapeseed flowers with different colors in the future.

The KEGG database is an effective tool for analyzing metabolic pathways and their interrelationships between groups, and the different metabolic pathways could be described in diagrams [43]. The differential metabolites of the flavonoid compounds in rapeseed petals with different colors were analyzed using the KEGG database to further characterize the interactions among the groups. The results of the KEGG annotation and enrichment analysis of the candidate differential metabolites suggested that the flavonoid biosynthesis, flavonoids’ and flavonols’ biosynthesis, and anthocyanin biosynthesis pathways played a pivotal role in the coloration process of rapeseed petals with different colors, which was in agreement with the results of a previous report [44]. These results have confirmed to some extent that the acylation and glycosylation of anthocyanins might have the greatest effect on the purple color of rape petals. In order to fully elucidate the main differential pigment accumulation pathways in rapeseed petals with different colors, we will identify candidate genes that contribute to the synthesis of coloring substances based on an homology search, transcriptome analysis, and ectopic expression in future experiments. Future experiments could use the current high-quality reference genome sequence to use these findings to explore the coloration mechanism of rapeseed petals with different colors.

The colorful rapeseed petals have the characteristics of deep color, high brightness, and bright color, of which its ornamental value has been comprehensively upgraded to promote the sustainable development of the rape flower sea tourism industry. And, the investigation of anthocyanidins is the basis of flower color improvement, and the clarification of the different coloring substances between white, yellow, pink, purple, and red petals in rapeseed could make the colorful rapeseed flower breeding work more directional. Through our study, it was found that the main differential pigments in the rapeseed petals with white and yellow colors were composed of flavones and flavonols, and the differential pigments between red and purple rapeseed petals are anthocyanins, flavones, flavonols, etc. Therefore, our research will not only be helpful to advance our knowledge of the characteristic constituents in rapeseed petals with different colors, but will also lay a firm foundation for breeding fine rapeseed cultivars with specific colors.

## 4. Materials and Methods

### 4.1. Materials

**Figure 6:**
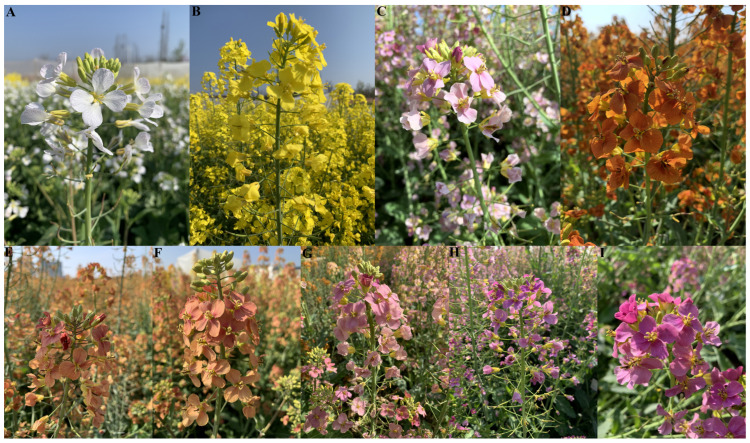
Petals colors of nine rapeseed cultivars lines: (**A**) white (W), (**B**) yellow (Y), (**C**) pink (P), (**D**) dark red (DR), (**E**) wine red (WR), (**F**) watermelon red (WMR), (**G**) light purple (LP), (**H**) purple (PP), and (**I**) dark purple (DP).

Nine rapeseed cultivars with different-colored petals, namely white (Figure 6A), yellow (Figure 6B), pink (Figure 6C), dark red (Figure 6D), wine red (Figure 6E), watermelon red (Figure 6F), light purple (Figure 6G), purple (Figure 6H), and dark purple (Figure 6I), were cultivated in the experimental field with similar soil conditions in Hantai district (Hanzhong, China). The nine rapeseed cultivars in this study were ‘White line’, ‘Yellow line’, ‘Pink line’, ‘Hanzi No.1’, ‘Ziluolan’, ‘Dark purple line’, ‘Wine Red line’, ‘Watermelon Red line’, and ‘Dark Red line’ (Table S7). Each rapeseed cultivar’s line was cultivated in 10 columns with 200 plants per column. All the rapeseed petal samples were collected at full flowering stage on 21 March 2023, put into liquid nitrogen, and then stored at −80 °C for differential coloring substance determination.

### 4.2. Petal Samples Preparation and Extraction

The fresh rapeseed petals samples were pulverized using a mixer (MM 400, Retsch, Haan, Germany) at 30 Hz with 1.5 min after being dried through a freeze dryer. In total, 100 mg of petal sample powder was added to 1200 μL of −20 °C pre-cooled 70% methanolic aqueous for extraction, and then the extracts were vortexed 6 times. The petal extracts were centrifuged (12,000 rpm at 4 °C for 3 min), and then all the supernatants were filtered with a micron microporous membrane (0.22 μm, ANPEL Laboratory Technologies, Shanghai, China), and were stored in the sampling bottles for UPLC-HESI-MS/MS analysis.

### 4.3. UPLC-HESI-MS/MS Conditions

The petal sample extracts were analyzed through an UPLC-HESI-MS/MS system (UPLC, ExionLC™ AD) and Tandem mass spectrometry system. UPLC was performed with a SB-C18 column (1.8 µm, 2.1 mm × 100 mm, Agilent, Santa Clara, CA, USA), with two mobile phases that were composed of solvent A (pure water with 0.1% formic acid) and solvent B (acetonitrile with 0.1% formic acid). Each extract sample was injected with 2.0 μL, and the flow velocity was set at 0.35 mL per minute at the column oven of 40 °C. The extract samples were performed with an elution gradient program: 9:1 solvent A: solvent B at 0 min; 1:19 solvent A: solvent B from 0 to 9 min; 1:19 solvent A: solvent B at 10 min; and 19:1 solvent A: solvent B from 11 to 15 min.

The parameters of the ESI source were as follows: the temperature of ESI was set to 550 °C; the positive ion mode (P+) was set to 5500 V; the negative ion mode (N−) was set to −4500 V; the GSI was set to 50 psi; the GSII was set to 60 psi; the curtain gas was set to 25.0 psi; the collision-induced ionization parameter was set to high; each ion pair was scanned and detected according to the optimized declustering potential and collision energy in the triple quadrupole.

### 4.4. Quantitative Analysis of Metabolites

Based on the self-built MVDB V2.0 database and metabolite information public database of Met Ware (Wuhan, China) Biotechnology Co., Ltd., the qualitative analysis of metabolites was performed using the secondary spectrogram information, and the quantitative analysis of each metabolite was completed using MRM analysis of triple quadrupole mass spectrometry. And then, the mass spectrogram of all metabolites in different-colored petal samples were obtained and the peak area of the mass spectrogram of each metabolite was integrated. Analyst 1.6.3 software (AB SCIEX, Concord, ON, Canada) was applied to process the UPLC-MS/MS data. Qualitative and quantitative analysis of each metabolite in nine rapeseed petals with different colors was analyzed using the local metabolite databases built by Met Ware. The chromatographic peaks’ integral and peak area correction of the mass spectrometry data were performed using MultiaQuant software (AB SCIEX, Concord, ON, Canada). The peak areas of each chromatogram peak represented the relative content of the corresponding metabolites.

The screening standards for differential metabolites were that the fold change in metabolite contents among the control groups and the experimental groups was not less than 2 (up-regulated) or not more than 0.5 (down-regulated), and the metabolites with VIP ≥ 1 were believed to be the statistically significant different between the control groups and the experimental groups. Through searching the KEGG (Kyoto Encyclopedia of Genes and Genomes) database, functional annotation analysis and metabolic pathway enrichment analysis were performed on metabolites with significantly different contents obtained from metabolomics analysis.

### 4.5. Statistical Analysis

The data for all metabolites were repeatedly collected 3 times, and all the data results were expressed as mean ± standard deviation (SD). Statistically significant differences among the nine rapeseed lines with different colors were assessed with analysis of variance (ANOVA) in SPSS v26.0 for Windows (SPSS Inc., Chicago, IL, USA) and Duncan’s test, and the *p*-values < 0.5 represented that the difference among groups was significant. Heatmap analysis, scatter matrix, violin plot, and histogram were drawn by using the Origin Pro 2023b for statistical computing (OriginLab, Northampton, MA, USA).

## 5. Conclusions

In this study, metabolite profiles were applied to investigate the variations in flavonoid and anthocyanidin contents in nine rapeseed petals with different colors using the UPLC-HESI-MS/MS approach. Our findings suggested flavones and flavonols were the main contributors to white and yellow petals, such as luteolin, quercetin, and kaempferol. And, tamarixetin-3-*O*-rutinoside, quercetin-3,7-Di-*O*-rhamnoside, butin-7-*O*-glucoside, and naringenin-7-*O*-glucoside were the main compounds in white petals. Tamarixetin and 4′-hydroxy-2,4,6-trimethoxydihydrochalcone were the main accumulated substances in yellow petals. Peonidin-3-*O*-(6″-*O*-caffeoyl) glucoside, peonidin-3,5-*O*-diglucoside, quercetin-4′-*O*-glucoside, and quercetin-7-*O*-glucoside were the main coloring substances in the pink petals. Acylation and glycosylation anthocyanins may be the main reasons for the difference in the formation of purple and red petals. Acylated cyanidin derivatives might contribute to a variety of purple colors. Glycosylated anthocyanins were responsible for the coloration of red rapeseed petals, and pe-onidin-3-*O*-glucoside and kaempferol derivatives were mainly detected from the red petals. Our study provided profound and comprehensive information on the chemical substances with different color rendering and different metabolic pathways in rapeseed petals with different colors, and the different candidate substances screened were conducive to the selection and breeding of colored rapeseed flowers with a specific color, which would have a great help in promoting the development of tourism in China.

## Data Availability

The datasets generated or analyzed in the current study are available from the corresponding author on reasonable request.

## Supplementary Materials

The following supporting information can be downloaded at: https://www.mdpi.com/article/10.3390/molecules28155670/s1, Table S1: Detailed information on the 543 metabolites identified and detected in nine rapeseed petals with different colors; Table S2: Differentially accumulated metabolites between W and Y petals; Table S3: Differentially accumulated metabolites between W and P petals; Table S4: Differentially accumulated metabolites between LP, PP, and RP petals; Table S5: Differentially accumulated metabolites between WR, WMR, and DR petals; Table S6: Differentially accumulated metabolites between W, Y, DP, and DR petals; Table S7: The petal color and genetic background of nine rapeseed cultivars; Figure S1: PCA score plot of the metabolite profiles between W, Y, P, LP, PP, DP, WR, WMR, DR, and QC (quality control); Figure S2: Heatmap analysis of differentially accumulated metabolites in W and Y petals. The color from blue (low) to red (high) indicates the level of each metabolite. The Z score represented the deviation from the mean in standard deviation units; Figure S3: The Veen plot of LP_vs_PP, LP_vs_DP, and PP_vs_DP (Figure S3A). The Veen plot of WR_vs_WMR, WR_vs_DR, and WMR_vs_DR (Figure S3B).
