# Supplementary material for: Characterization Variation of the Differential Coloring Substances in Rapeseed Petals with Different Colors Using UPLC-HESI-MS/MS
Source: Molecules. 2023 Jul 26;28(15):5670. doi: 10.3390/molecules28155670 (PMC10419860; doi:10.3390/molecules28155670)
Supplement: Supplementary file 1 [file molecules-28-05670-s001.zip › Table S7.pdf]

Table S7. The petals color and genetic background of nine rapeseed cultivars

Rapeseed cultivars	Petals color	Genetic background
'White line'	White	'*Brassica napus* L. ZS-1'×'*Brassica napus* L. 11-231'
'Yellow line'	Yellow	'*Brassica napus* L. Z11'×'*Brassica napus* L. Y4'
'Pink line'	Pink	'*Brassica napus* L. FD-1'×'*Brassica napus* L. 22-231'
'Hanzi No.1 line'	Light Purple	'*Brassica napus* L. 08-H16' ×'*Raphanus sativus* L. ZH-1'
'Ziluolan line'	Purple	'*Brassica napus* L. 08-H16' ×'*Raphanus sativus* L. ZH-3'
'Dark purple line'	Dark Purple	'*Brassica napus* L. 08-H16' ×'*Raphanus sativus* L. ZH-3'
'Wine red line'	Wine Red	'*Brassica napus* L. 08-H16' ×'*Raphanus sativus* L. ZH-3'
'Watermelon Red line'	Watermelon Red	'*Brassica napus* L. QY211R' ×'*Brassica napus* L.16-002'
'Dark Red line'	Dark Red	'*Brassica napus* L. QY211R' ×'*Brassica napus* L.16-002'

Note: All the rapeseed lines were selected by hybrid breeding.
