# Supplementary figures and images for: Characterization Variation of the Differential Coloring Substances in Rapeseed Petals with Different Colors Using UPLC-HESI-MS/MS
Source: Molecules. 2023 Jul 26;28(15):5670. doi: 10.3390/molecules28155670 (PMC10419860; doi:10.3390/molecules28155670)

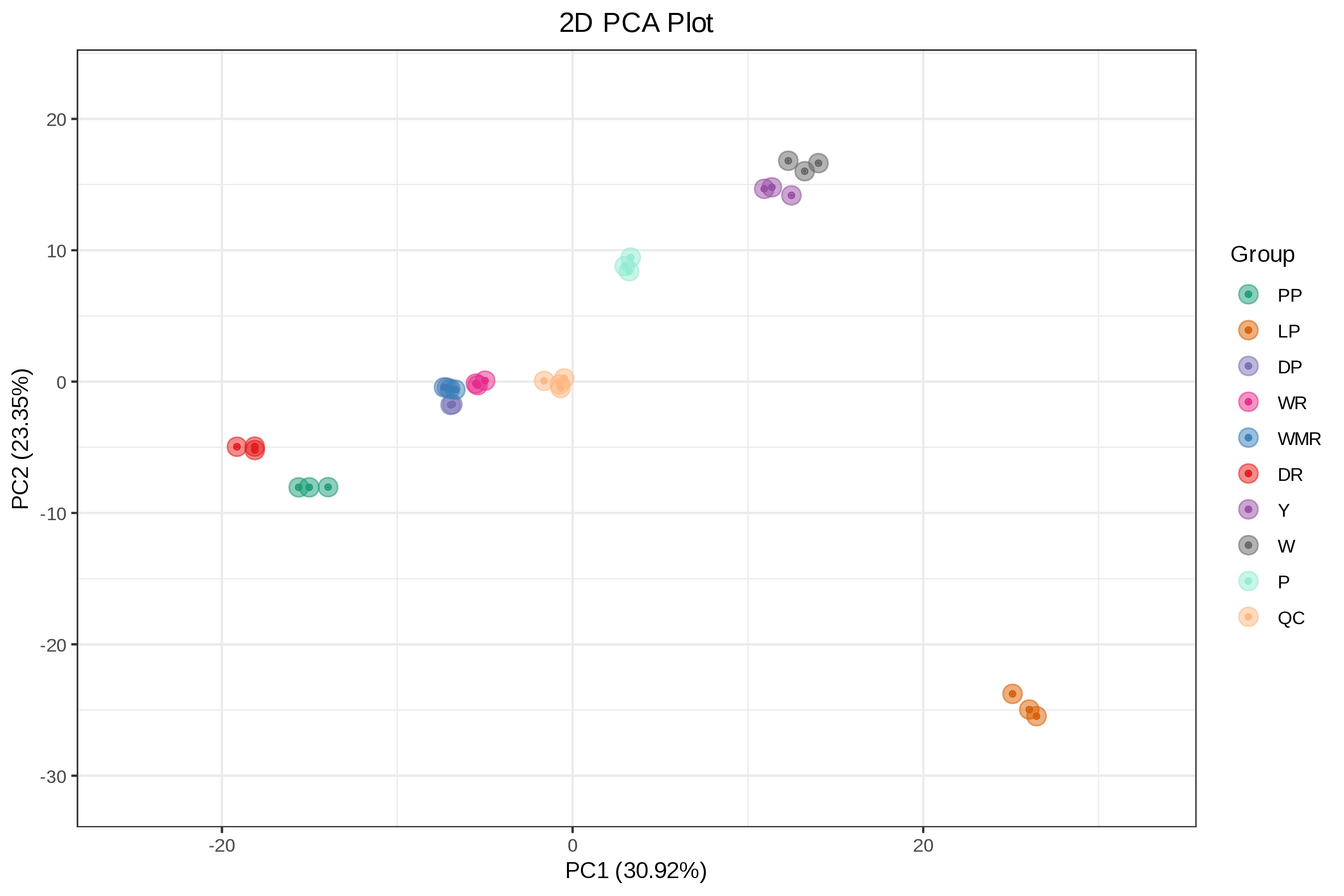

Supplement: Supplementary file 1 [file molecules-28-05670-s001.zip › Fig. S1.png]

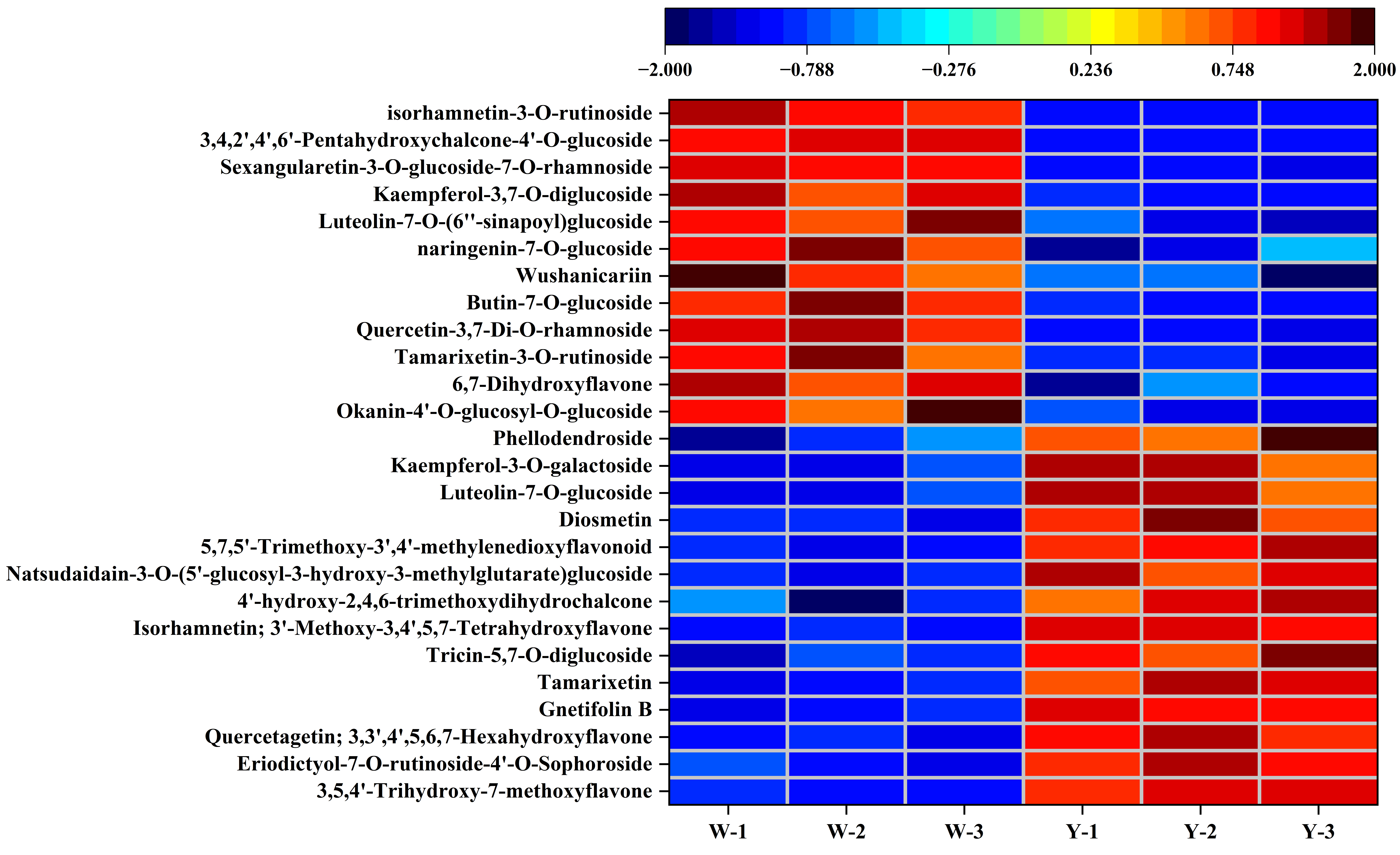

Supplement: Supplementary file 1 [file molecules-28-05670-s001.zip › Fig. S2.jpg]

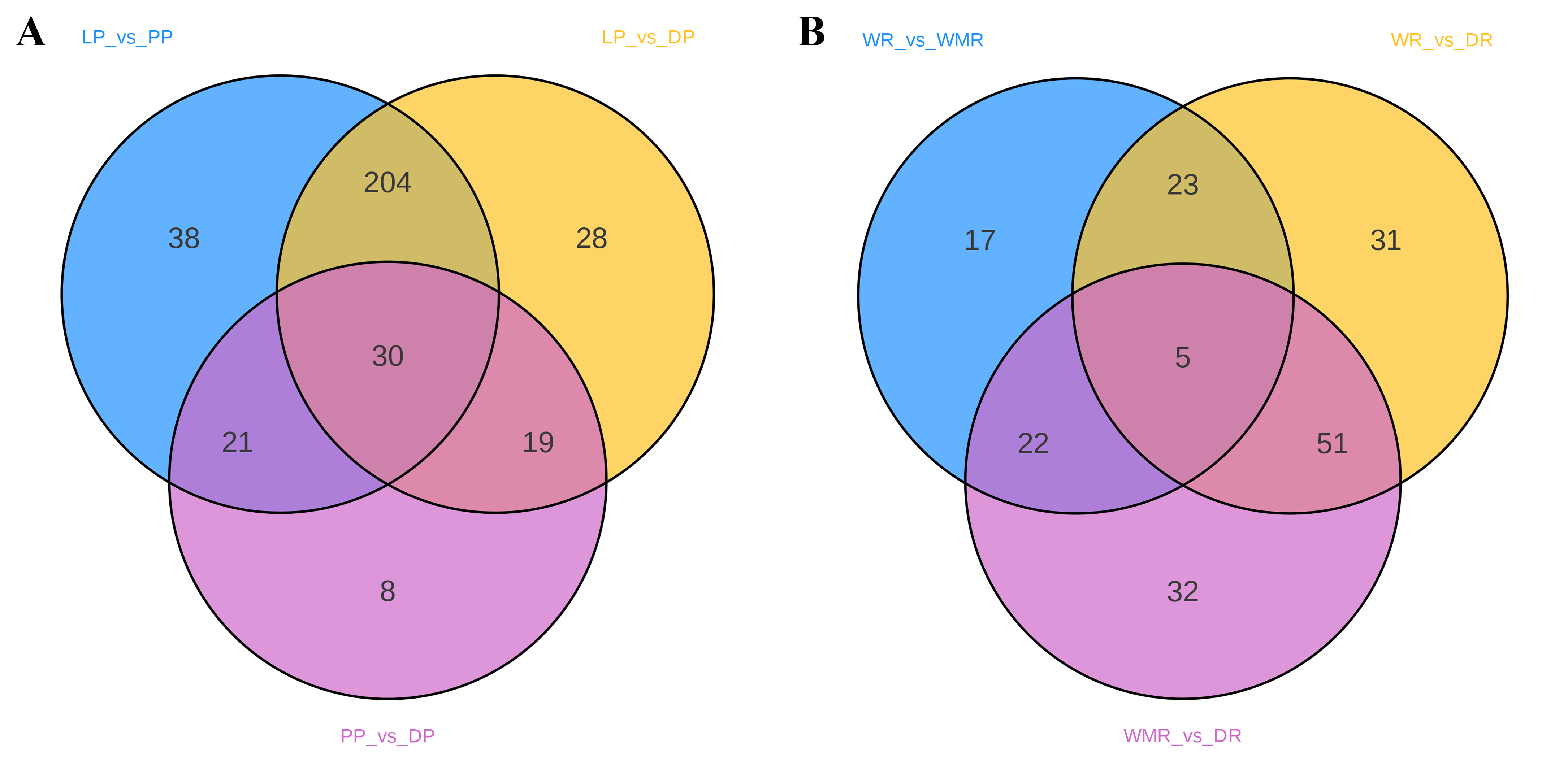

Supplement: Supplementary file 1 [file molecules-28-05670-s001.zip › Fig. S3.jpg]
